# Supplementary material for: Twisted bilayer zigzag-graphene nanoribbon junctions with tunable edge states
Source: Nat Commun. 2023 Feb 23;14:1018. doi: 10.1038/s41467-023-36613-x (PMC9950076; doi:10.1038/s41467-023-36613-x)
Supplement: Supplementary file 1 — Supplementary Information [file 41467_2023_36613_MOESM1_ESM.pdf]

Supplementary Information: Twisted bilayer zigzag-graphene nanoribbon junctions with tunable edge states

Dongfei Wang[1,*], De-Liang Bao[1,*], Qi Zheng[1], Chang-Tian Wang[1], Shiyong Wang[2], Peng Fan[1], Shantanu Mishra[2], Lei Tao[1], Yao Xiao[1], Li Huang[1], Xinliang Feng[3,4], Klaus Müllen[5], Yu-Yang Zhang[1], Roman Fasel[2], Pascal Ruffieux[2] ✉, Shixuan Du[1]✉, and Hong-Jun Gao[1]✉

[1]*Institute of Physics & University of Chinese Academy of Sciences, Beijing 100190, China.*
[2]*Nanotech@surfaces Laboratory, Empa, Swiss Federal Laboratories for Materials Science and Technology, Dübendorf, Switzerland.*
[3]*Center for Advancing Electronics Dresden (cfaed) and Faculty of Chemistry and Food Chemistry, Technische Universität Dresden, 01062 Dresden, Germany*
[4]*Max Planck Institute of Microstructure Physics, Weinberg 2, 06120 Halle, Germany*
[5]*Max Planck Institute for Polymer Research, 55128, Mainz, Germany*
**These authors contributed equally: Dongfei Wang, De-Liang Bao.*
✉e-mail: pascal.ruffieux@empa.ch; sxdu@iphy.ac.cn; hjgao@iphy.ac.cn

TABLE OF CONTENTS

- Supplementary Notes 1-9
- Supplementary Figures 1-13
- Supplementary References

Supplementary Notes:

1. **Reversible STM Tip Manipulation of ZGNRs**

It is reported that the graphene nanoribbon with armchair edge has superlubricity on gold surface[1]. Here, by STM tip manipulation, we proved that the graphene nanoribbon with zigzag edge can also be easily lateral moved and manipulated on gold surface like the arm-chair case[2]. As shown in Supplementary Figure 2(b), the ribbon was firstly bent by the STM lateral manipulation in a direction indicated by the white arrow. Then, the ribbon was bent back to its original position with tip manipulation in a reversal direction as shown in 2(c) and 2(a). Furthermore, we bent the ribbon again in the direction same with 2(b) but with a smaller bending angle as shown in 2(d). From 2(a) and 2(c) we can see the ribbon has similar quality after the bending.

2. STM images of more TBZGNR Junctions

With the STM lateral tip manipulation technique mentioned above, we can achieve TBZGNR junctions with different twist angles. 16 of them are shown in Supplementary Figure 3. The achieved twisted angles range from 30° to 90°.

3. Charge redistribution within the TBZGNR junction

Previous study on bilayer graphene[3] shows the finite interlayer hopping could influence the low-energy band structure of graphene. This is also true for the case of bilayer graphene nanoribbon. The interlayer hopping manifests itself as a finite bonding between the electrons of the top and bottom ribbon. Thus, the effective electron charge redistributes in the overlapping region. By DFT calculation, this effect is clearly shown in Supplementary Figure 4 where a net electron charge accumulation happened in the space between the top and bottom ribbon.

4. DFT results for another TBZGNR structural model, Model D

In order to further verify the theory that a TBZGNR without symmetry can support spin polarized flat band at the edge as we argued in the main text and Figure 4(e), we construct another TBZGNR structure model D also without symmetry. The DFT calculated results are shown in Supplementary Figure 5. From Supplementary Figure

5(b) we can see again a pronounced peak near zero energy is developed only near the corner of the overlapped region. This peak also originates from a new flat band near zero shown in 5(c). We can further identify the bands are spin non-degenerate with a relatively small splitting energy. All these features are similar to what we obtained for the model C in the main text. Thus, our argument that the asymmetrical van de Waals potential produces spin polarized flat bands in TBZGNR is repeated in another model structure.

5. Influence of the Au (111) surface state and step edge on the spectra of monolayer ZGNR

As the ZGNR used in this work is the same as that used in the previous reference[4], the edge only terminated with C-H bond. Because we do not intentionally make a bias pulse, the C-Au bonding reported in the other reference[5] is also not the case. From the previous theory prediction[6] it is shown that the ZGNR on Au (111) still displays a magnetic edge state with antiferromagnetic coupling between the edges. The magnetization per edge C atom is about 0.22 μB which is comparable to the free-standing ZGNRs. These edge states are not observed mainly due to the strong extension of the surface state of Au (111) in the out of plane direction. From the Figure 2 in previous report[7] we can clearly see the Au (111) surface state survive even 2 Å away from the surface. The apparent height of our monolayer ZGNR is just 1.85 Å. Thus, most of the ZGNR edge states are in the shadow of the Au (111) surface state, this is also the reason why the DOS at the monolayer edge mimics the surface state of Au (111), as already seen in the Figure 2h.

Recently, an interesting work[8] reported effective pi bonding between C and Au atoms when the ribbon edge is doped by nitrogen. However, this is not the case here as we never see an effective decoupling of the monolayer ribbon by ramping a bias sweep, also the ribbon used is not nitrogen doped.

As the bottom ribbon of the junction always lies in the vicinity of the Au (111) step edges, it is a necessary to discuss the influence of the step edges on the density of state we obtained on the TBZGNR junction. Taking one junction with a twist angle near

80° for example, the Au step edge is highlighted with a white dashed box in the STM topography image shown in Supplementary Figure 7(a). By comparing with corresponding dI/dV mapping images at energies we interested in this study, we did not find any evident density of state distribution on the Au step edges, as shown in 7(b) and 7(c). In contrast, the edge state of the top zigzag graphene nanoribbon at the junction is clearly demonstrated, as highlighted by the yellow arrows. Thus, it concludes that the step edge of the Au (111) will not give additional influence on our data analysis regarding the energies we are interested.

6. Exclusion of out-of-plane bending and lattice distortion effects

We estimate the out-of-plane bending effect of the top ZGNR by taking the line profile across the TBZGNR junction, as demonstrated in Supplementary Figure 10(a). From the results shown in 10(b) we can see that the ribbon bending angle $\theta=165.3^o$, which means very tiny bending and out-of-plane distortion. Our theoretical calculation also gives a similar bending angle near 177° which is even larger than the value measured from the experiment. The previous theoretical calculations indicates that when the bending angle θ range between 100° and 180°, both the AFM magnetic ground state and the energy gap are essentially the same[9]. Thus, the pure bending and out-of-plane distortion effect will not play a role in our study.

On the other hand, the lattice distortion in graphene system can also introduce some important physical effect such as pseudo-magnetic field. Compared to the previous reports where the strain mainly due to a designed structure confinement by the substrate[10,11], the fabrication process of TBZGNR does not introduce any additional strain, as: 1) the Au surface is flat and no substrate template effect; 2) the end of the top ribbon is free, strain due to confinement can be ignored. There are some possibilities that the Van der Waals and gravity can introduce some lattice distortion at the TBZGNR junction edge, However, from some simple calculations we can show that these two effects can also be excluded, as shown below:

We consider two extreme cases:

a) the lattice distortion only induced by the gravity of the tail part of the top GNR as illustrated in Supplementary Figure 10(c). If we assume the tail part has a length of 5 nm, in total we have 264 carbon atoms there, which has a gravity of 5.17×10^{-23} N. The

cross section at the edge of the junction is 5.1×10^{-19} m2 if we take the interlayer distance 0.45 nm and the width of the ribbon 1.14 nm. Therefore, the stress at the edge is 1.01×10^{-4} Pa. Because of the Graphene´s super high Young's modulus of E = 1.0 TPa, the strain at the edge is 10^{-16}, which is very tiny.

b) the lattice distortion only induced by the in-plane van de Waals´ pulling force of the tail part of the top GNR, as illustrated in Supplementary Figure 10(d). From previous experiment[12] we learned the shear stress of graphene on a silicon oxide surface is around 1.64 MPa, if we take the same Young´s modulus 1 TPa, we get a strain of 1.64×10^{-6}, which is also very small.

It is demonstrated experimentally that 1%-2% strain in graphene can only introduce 0.7 T pseudo-magnetic field[13]. So, in our case there is no additional strain and related pseudo-magnetic field induced by both gravity and van der Waals force. This is also demonstrated in Figure 3 that no corresponding symmetric Landau Levels found in experiment and DFT calculation.

There is another evidence that the near zero energy bound state does not originate from strain effect. The dI/dV mapping image at -40 mV in main text Figure 2g shows the bound state localized at both the edges and the corners of the junction. The periodicity is the same as that of the zigzag carbon atoms, which demonstrate the near zero energy bound state is junction structure related other than strain related.

7. Exclusion of supercell size and a possible intersupercell interaction effect

The total energy per atom as a function of the lattice constant of the supercell were carefully tested in the calculation. As shown in the Supplementary Figure 8(a) and 8(b), the energy difference converges to 0.001 eV/atom from L≈27 Å to 32 Å. We chose the 27 Å cell (11 carbon rows in length) to do all other calculations, considering the balance between calculational performance and cost. The calculated electronic structures of modeled TBZGNRs already agree well with experiments for L≈27 Å.

We further did a calculation on a non-periodic structure for comparison as shown in Supplementary Figure 8(c) and 8(d). From the projected density of states (PDOS) shown in 8(d) we can see the results are highly similar with that in the main text Figure 3f, which demonstrate that the featured asymmetric PDOS peaks originate from the stacking.

We also checked the PDOS at the edge of monolayer ZGNR in the vicinity of the TBZGNR junction with DFT calculation, as shown in Supplementary Figure 9. The PDOS at the monolayer ZGNR edge already demonstrating a gap like line shape, mimic that of the pristine GNR, although the corresponding carbon atom just shift 2 lattice constants from the junction corner. The featured near-zero-energy peaks observed on the edge atoms in the crossing region are obviously absent.

Thus, the possibility that the emergence of the near-zero-peak owing to the calculating size of the supercell and possible interactions between periodic supercells are excluded.

8. Exclusion of the influence of bright protrusions on the edge state

As there are some bright protrusions on Figure 2f and 2j, it is a necessary to check they has no relation with the edge states we discuss in the paper. By checking the dI/dV mapping in Fig. 2g we found no additional signal belongs to the protrusion shown in Fig. 2f at the edge state energy -40 mV. From the tip manipulation of the top ribbon shown in Fig. 2i-2k, we learned the bright feature on the right edge of top ribbon appears for the first manipulation (Fig. 2j) and disappears again (Fig. 2k) after manipulating back. As our tip is far from the junction during the manipulation, it is very unlikely to be adatom. These bright protrusions are also excluded to be "mouse-bite" type defect on the edge of the single layer ZGNR because of two facts. Firstly, the bright protrusions shown in Figs. 2f,j and Fig. 3b appear in the middle of the top ribbon edge within the junction, which means they sit on the middle of the bottom ribbon. However, the "mouse-bite" type defect appears mostly on the edge of the bottom ribbon. Secondly, as seen in Figure S4 in the supplementary information of reference 4, the STS on the "mouse-bite" type defect (indicated by red triangle) does not resemble what we have shown in Figure 3(a-c), where either clear gap feature or in-gap states were observed. Thus, we can safely exclude that the bright features in Figs. 2f,j and the insets for Fig. 3a,b are this type of defect. We propose the protrusions we observed to be a tiny stress states at the edges which, as we demonstrated already in previous section, will not lead to great impact (the top ribbon edge at the junction is still very straight and has a strain less than 1%).

9. Nc-AFM measurements on a 76° TBZGNR junction together with DFT calculation

To unambiguously make a link between the structure of the junction and the observed edge state, we employed both STM and nc-AFM and got some initial results regarding the structure and DOS. The new junction we studied has a twist angle of 76° as shown in Supplementary Figure 11a. In order to determine the atomic structure, nc-AFM measurements were done on both the bottom and top 6-ZGNR as shown in 11b and 11c. By extending the model structures from nc-AFM measurements we resolve the atomic model of this 76° TBZGNR junction shown in 11d. By comparing the experimental dI/dV spectra (Supplementary Figure 11f) with the DFT-calculated PDOS (Supplementary Figure 11g) along a similar path across the junction, one can get good agreement between the experiment and the calculation, just like the data shown in the main text Figure 3. For example, the peaks just above the Fermi energy are strongest only at the edge, and the signal at the left edge is slightly stronger than that at the right edge, as highlighted by the red dashed lines. The relative energy positions of the peaks below the Fermi energy (highlighted by black and blue arrows) to those just above the Fermi energy (highlighted by red dashed lines) also agree qualitatively between experiment and calculation. Noteworthy, the as-constructed 76° junction also lacks inversion or mirror symmetry, so asymmetric edges states were found both by experiment and calculation. Thus, the nc-AFM measurements further support our argument in the main text and emphasize the importance of stacking offset in the determination of edge state.

Supplementary Figures 1-13

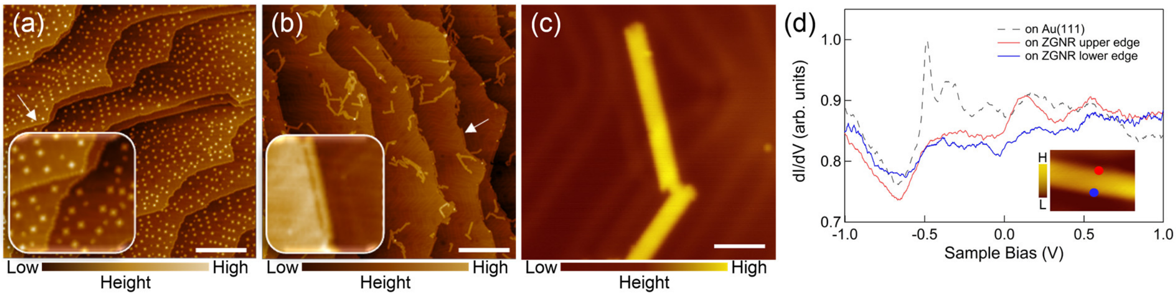

Supplementary Figure 1. Growth and characterization of individual 6-ZGNRs on Au (111). (a) STM topography image of the precursor deposition on Au (111) surface. Inset: Zoom in image of the precursor absorbed near the Au step edge. (b) STM topography image of ZGNR after annealing the pre-deposited precursors on Au (111) surface. Inset: Zoom in image of ZGNR formed near Au step edge. (c) STM topography image of the monolayer ZGNR formed on the Au terrace. (d) STS taken on the top (red) and bottom (blue) edge of the monolayer ZGNR. Grey curve shows a typical STS on the gold surface. Inset: STM topography image of a monolayer ZGNR indicating where the STS were taken, size 5.1 nm×3.6 nm. Scale bar: (a-b) 40 nm, (c) 4 nm. Tunneling parameters: (a-b) V=0.5 V, I=50.0 pA, (c) V=-0.3 V, I=1.0 nA; (d) V_{stab}=-0.3 V, I_{stab}=1.0 nA, V_{osc} =0.5 mV

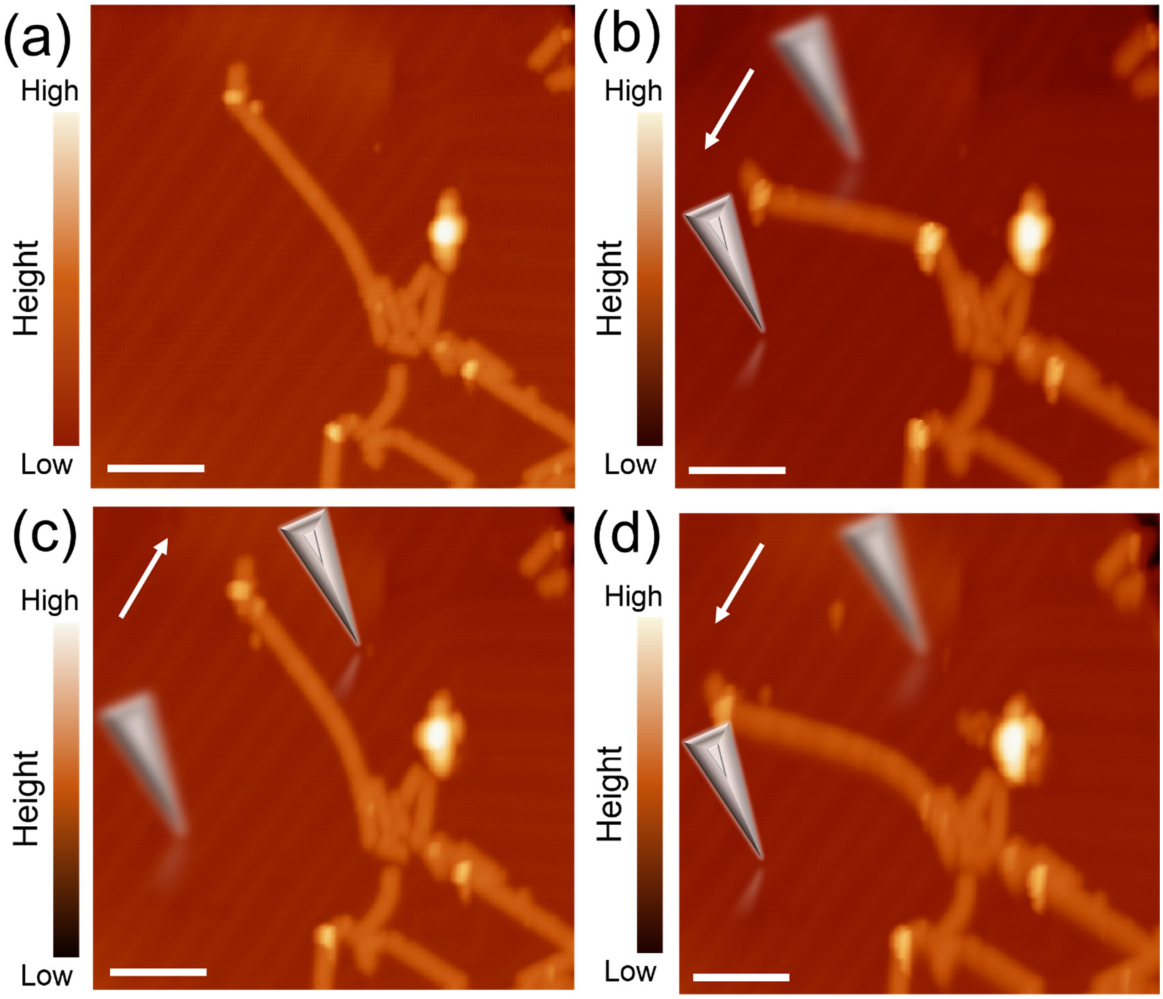

Supplementary Figure 2. Demonstration of STM tip manipulations on a monolayer ZGNR. (a-d) Reversible STM tip manipulation of ZGNR demonstrated with sequential STM topography images. The blurred and solid silver triangle illustrate the tip position before and after tip manipulation. The white arrow indicates the manipulating direction. Scale bar: (a-d) 10 nm. Tunneling parameters: (a-d) V=-0.3 V, I=1.2 nA.

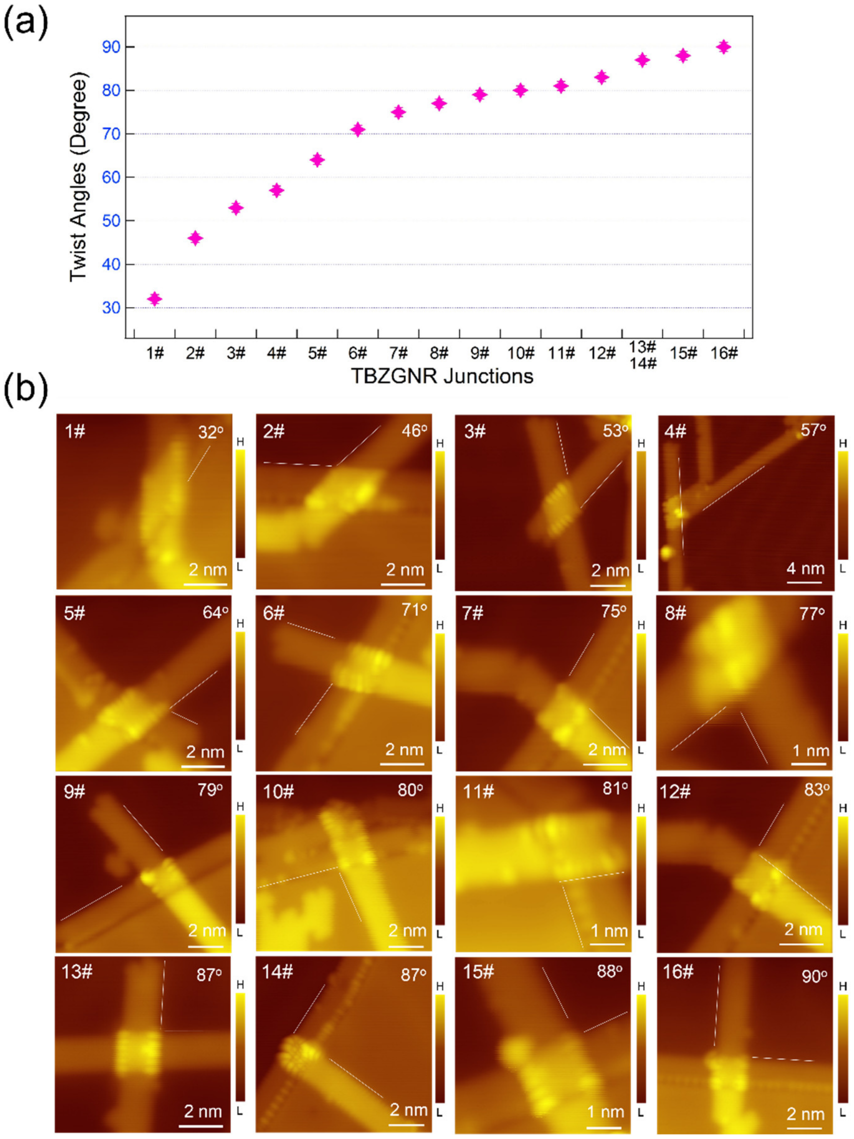

Supplementary Figure 3. **16 TBZGNR junctions prepared in this study.** (a) The twist angles distribution of the 16 as-fabricated TMZGNR junctions. (b) STM topography images of the 16 TBZGNR junctions with twist angles ranging from 32° to 90°.

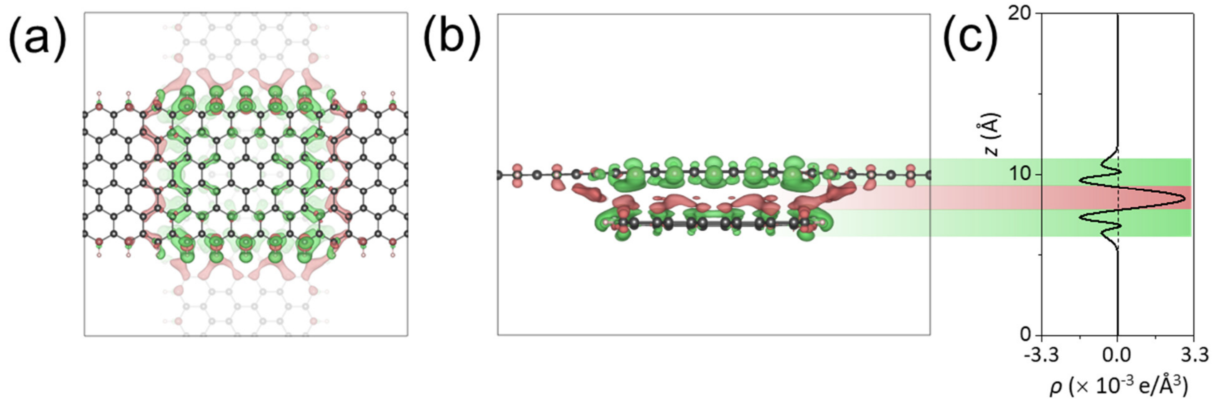

Supplementary Figure 4. Charge redistribution in the TBZGNR junction revealed by DFT calculation. (a-b) Top and side views of the charge density difference between the two overlapping ZGNRs. The charge density difference is obtained by $\rho_{diff}=\rho_{total}-\rho_{top}-\rho_{bottom}$, where ρ_{total} is the electron density of the TBZGNR, ρ_{top} and ρ_{bottom} are the electron density of only top or bottom layer ZGNR. Thus, the plots illustrate how the electrons evolve when two intrinsic ZGNRs meet. Green surfaces represent electron depletion and red surfaces represent electrons accumulation. It is clear that within the overlap region, electrons move out from the ZGNRs and accumulate in the space between them. (c) The xy-plane averaged charge density difference, where positive values mean electron gathering and negative values mean electron losing. With the help of the color shadow, one can find clearly that, spatially, in the two ZGNRs planes electrons decrease while in the middle plane electrons increase.

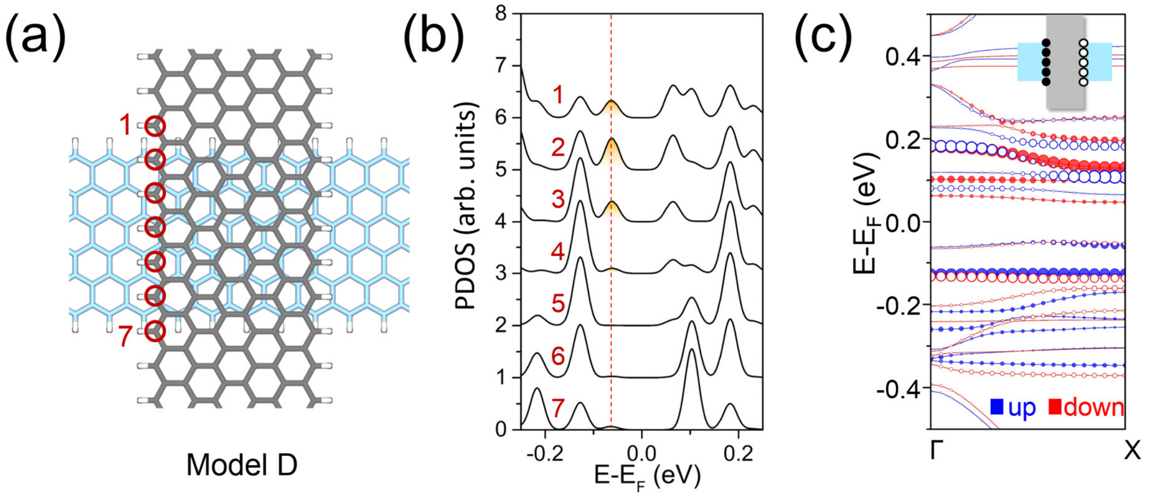

Supplementary Figure 5. DFT calculation results for another asymmetric junction model D. (a) Top view of the atomic model of another designed TBZGNR structure model D without symmetry in the overlapping area. The gray GNR is on the top. The light blue GNR is on the bottom. (b) PDOS on the edge carbon atoms labeled point 1 to point 7 in (a). The red dashed line indicates a pronounced bound state close to zero energy appears only in the regions near point 2 which is quite similar to what we calculated in model C in the main text. (c) Calculated band structure (solid lines) of the model D in (a). The solid/open circles represent projections on the left/right edge of the top GNR within the overlapping area, respectively. Blue and red colors correspond to spin up and down, respectively. One can find the spin-split bands again in this overlap region without lattice symmetry, which is similar to the case of the model C.

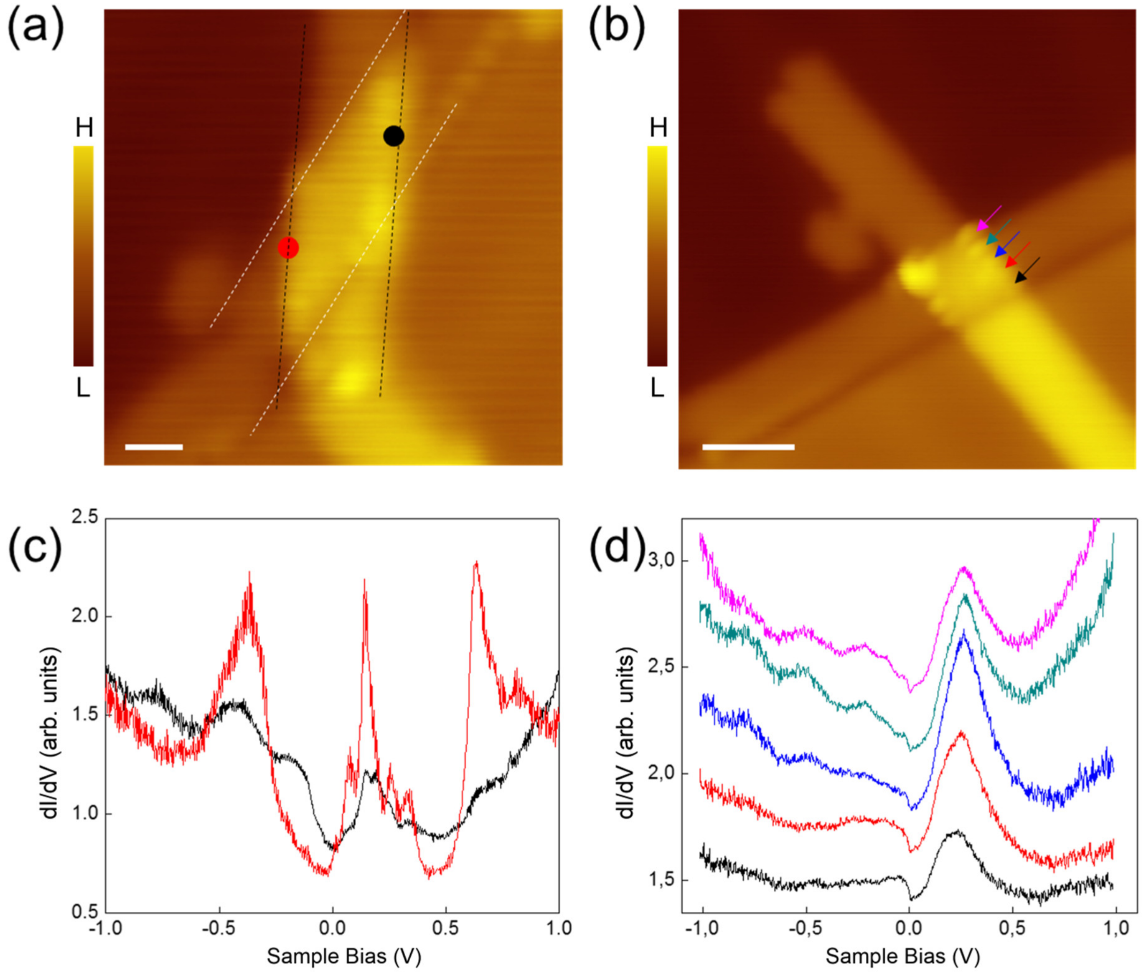

Supplementary Figure 6. STS on two TBZGNR junctions with different twist angles. (a) Topography image of the TBZGNR junction with $\theta = 32\circ$. The white and black dashed lines indicated the edges of bottom and top ZGNR. (b) Same as (a) but the twist angle is 79 ∘. (c) STS spectra taken on the edges of the overlap region shown in (a). Red/black spectrum recorded on the positions highlighted by the red/black dot. (d) STS spectra taken on the positions on the right edge of the TBZGNR junction indicated by the arrows. Scale bar: (a) 1 nm (b) 2 nm. Tunneling parameters: (a,b) V=-320 mV, I=1.0 nA; (c,d) V_{stab}=-320 mV, I_{stab}=1.02 nA

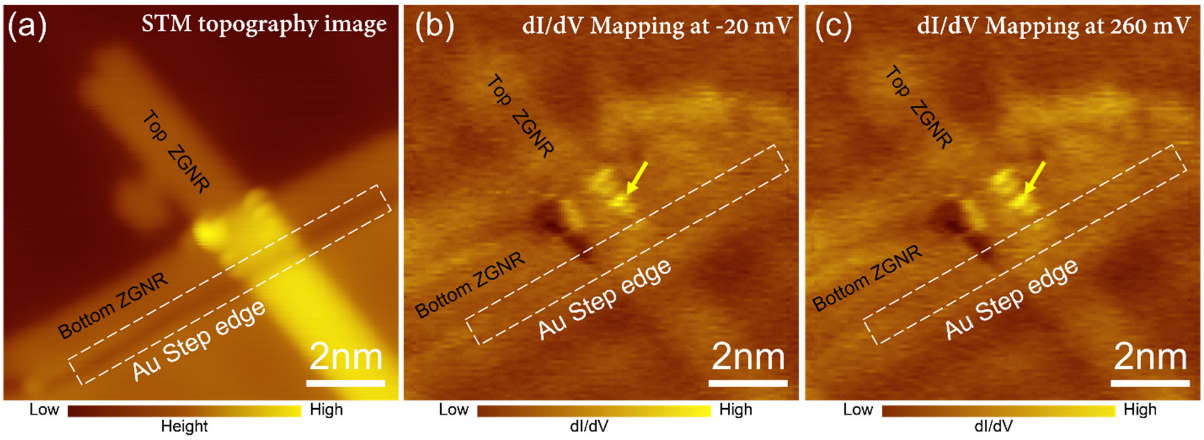

Supplementary Figure 7. dI/dV signals near the Au step edge. (a) STM topography image of one TBZGNR junction with twist angle 79°. The Au (111) step edge is highlighted with white box. (b,c) The dI/dV images of the same area at energies -20 mV and 260 mV correspondingly. The yellow arrow indicated the edge states of this TNZGNR junction.

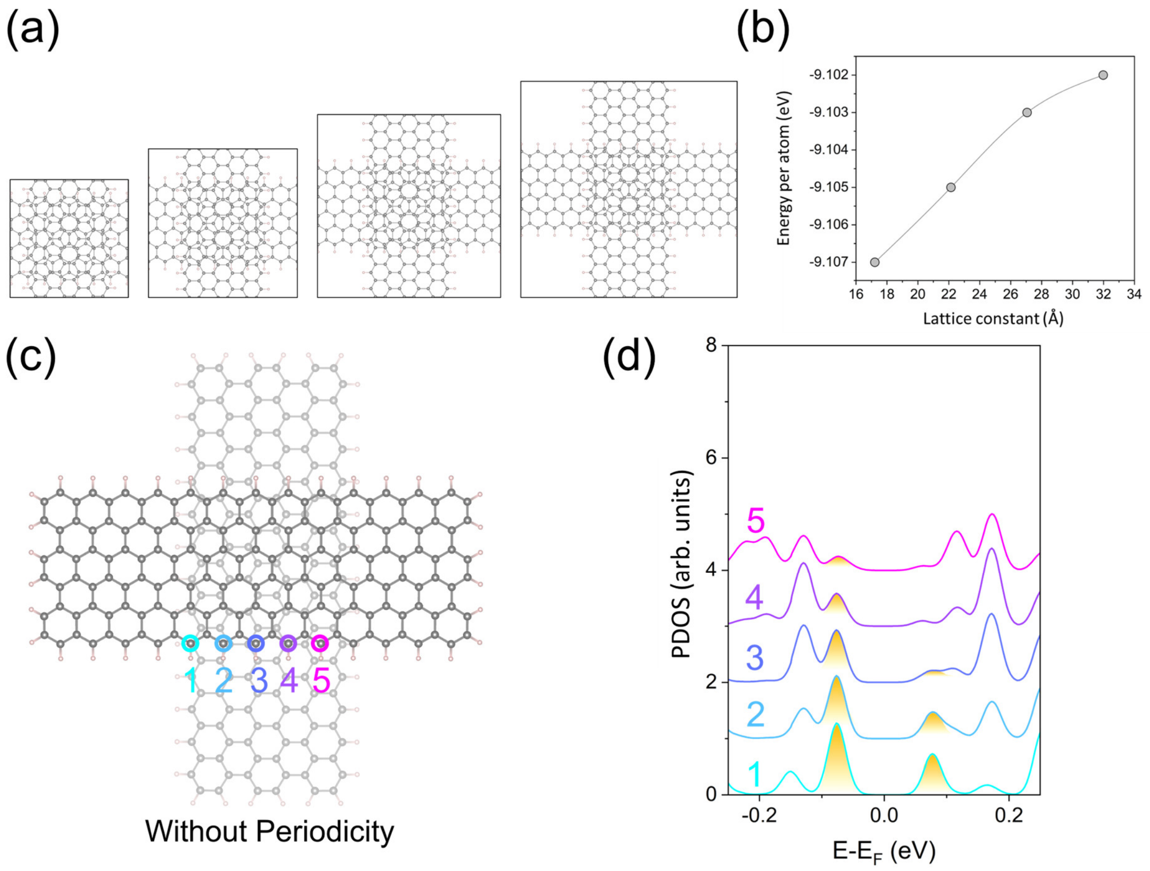

Supplementary Figure 8. DFT calculations for the TBZGNR model C under different boundary conditions. (a-b) Energy per atoms as a function of lattice constant of calculational cells. The cell sizes are 17 Å, 22 Å, 27 Å and 32 Å respectively. (c) The top view of the fragments model. The central overlapping region is the same as that in model C (Figure 3f). The five edge atoms that are projected on are highlighted with colorful circles. (d) The PDOS on the five highlighted edge atoms in (c).

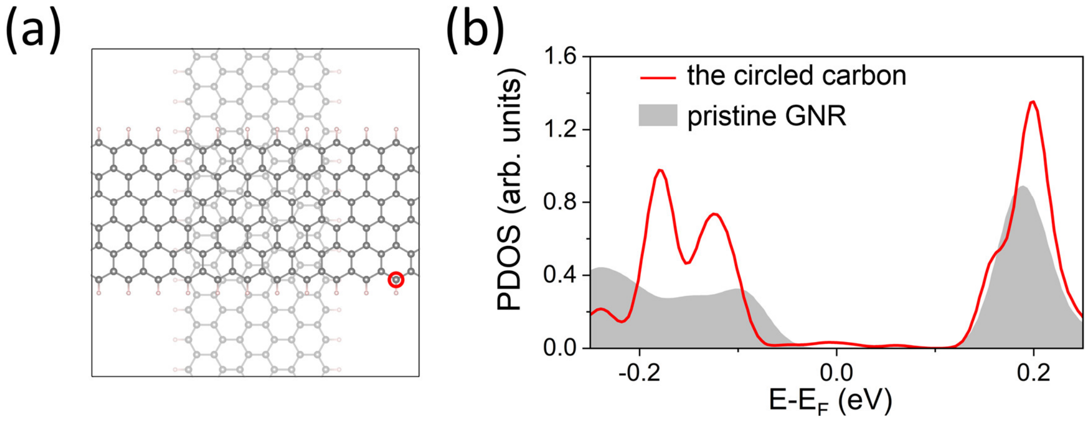

Supplementary Figure 9. DFT calculations for the monolayer ribbon edge atom close to the junction. (a) The top view of model C with a marked carbon that is off the overlapping region. (b) The projection density of state (PDOS) on the carbon in (a) (red curve) and the DOS of pristine GNR (dark shade).

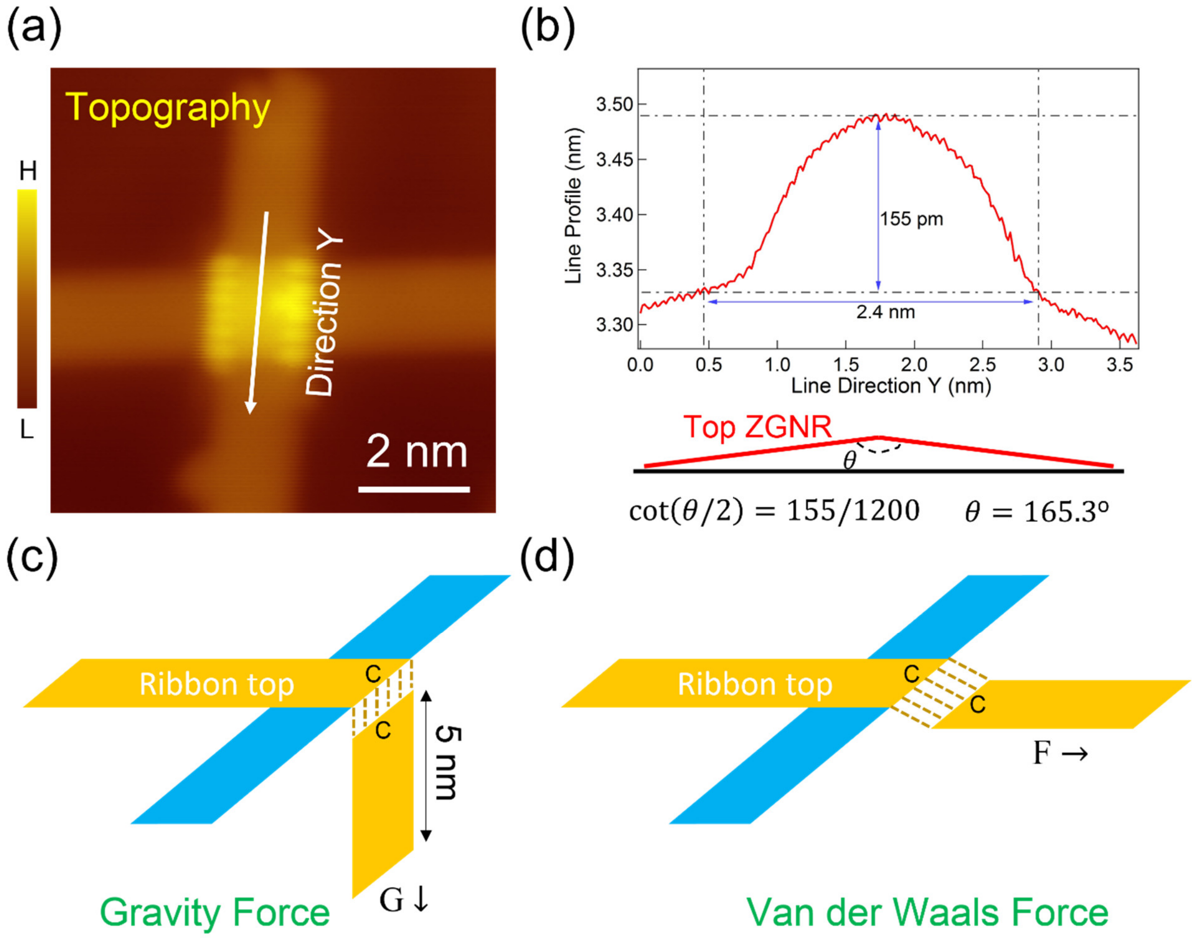

Supplementary Figure 10. **Strain effect in the top layer ZGNR.** (a) A replot of the STM topography image of the TBZGNR junction shown in Figure 2f. The white arrow indicates where the line profile is taken. (b) Upper panel: The line profile of the top ZGNR in the vicinity of the TBZGNR junction along the direction shown in (a). Lower panel: A simplified model plot showing the bending of the top ZGNR in the vicinity of the junction. The bending angle is 165.3^{o} according to the data obtained in (a). (c) and (d), Schematic diagram showing local lattice distortion of the top ZGNR by only gravity force (c) and Van der Waals force (d).

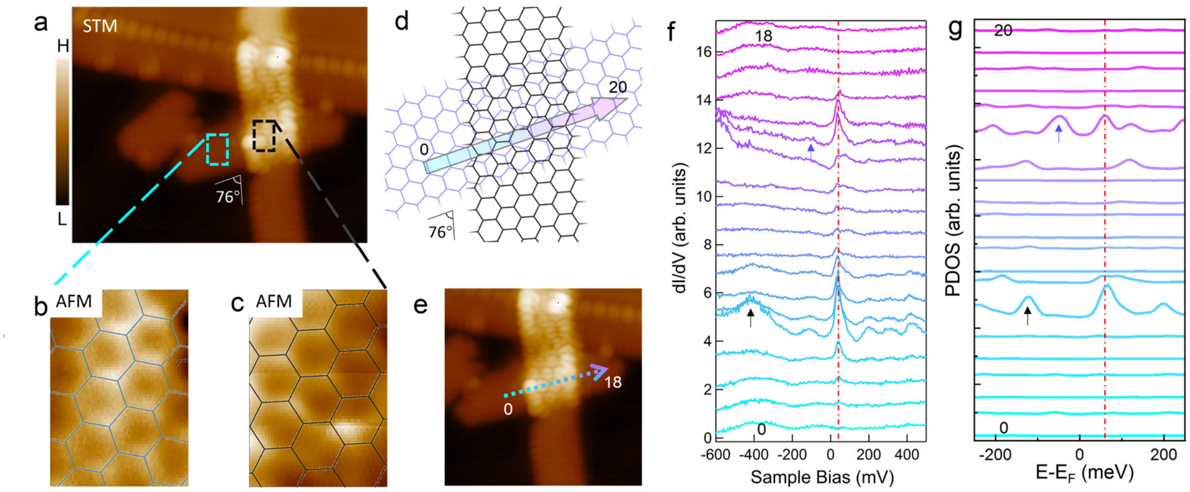

Supplementary Figure 11. AFM characterization and DFT calculation on a 76° TBZGNR junction. (a) The STM image of a TBZGNR junction with a twist angle of 76°. Size 7 nm×10 nm. (b, c) Zoom-in AFM images of the bottom ZGNR (light blue) and top ZGNR (black), respectively. Size 0.7 nm×1 nm. The models are superimposed on the images. (d) TBZGNR junction with the stacking configuration obtained by extending the AFM-measured structures in (b) and (c). (e, f) 19 dI/dV spectra (f) taken across the top ribbon edges along the arrow direction shown in (e). (g) DFT calculated PDOS on 21 atoms along the path (colorful arrow) shown in model (d). The red dashed lines in (f) and (g) highlight the edge states just above Fermi energy. Tunneling parameters: (a, e) V=-50 mV, I=10 pA; (f) V_{stab}=-50 mV, I_{stab}=30 pA.

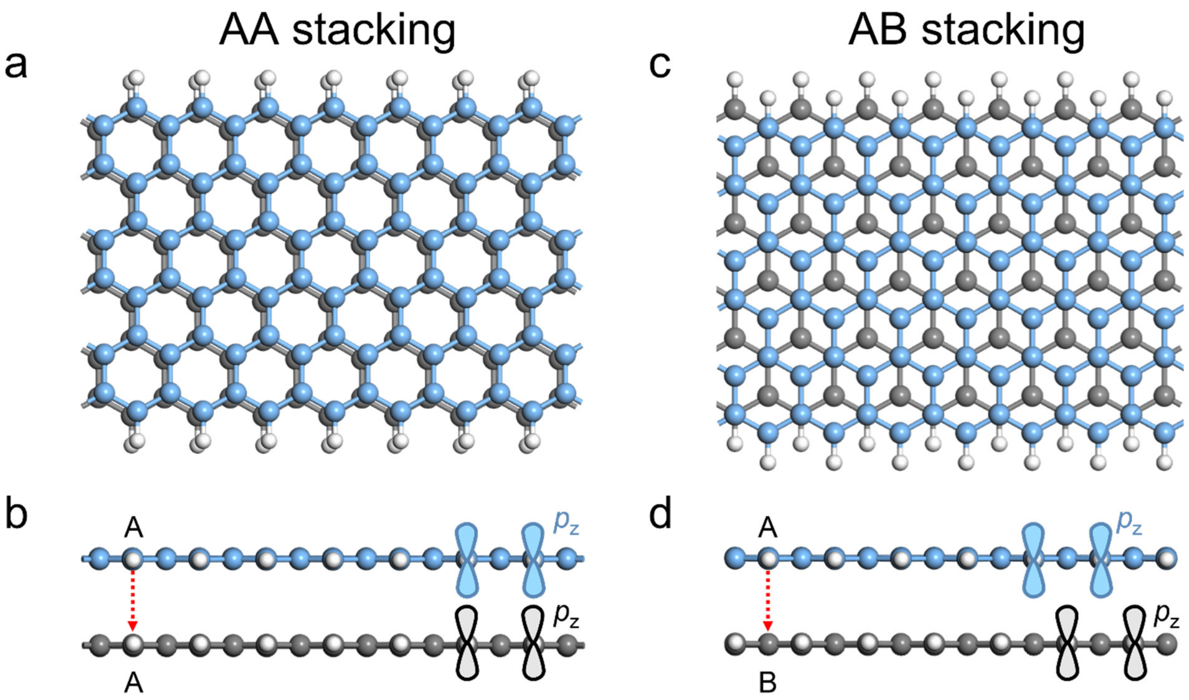

Supplementary Figure 12. **Model structures for AA- and AB-stacking bilayer ZGNRs.** (a, b) Top-view and side-view of AA-stacking bilayer GNRS geometry. (c, d) Top-view and side-view of AB-stacking bilayer GNRS geometry. Grey: bottom ribbon, Blue: Top ribbon. In the top view of the AA-stacking, the top ribbon is shifted a bit for clear visualization. The p_z orbitals of edge carbon atoms were illustrated to help recognizing the atomic stacking on the edges.

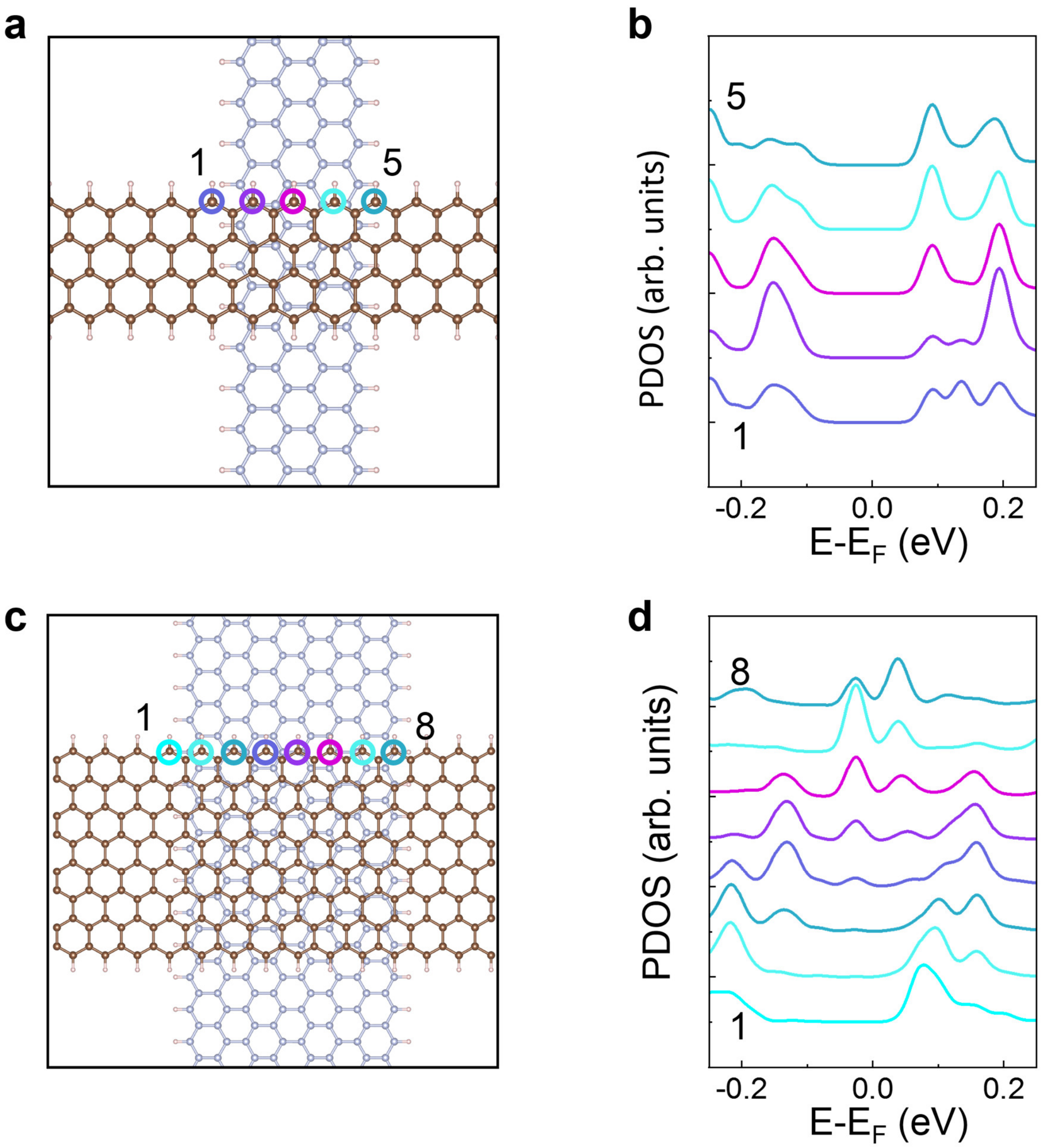

Supplementary Figure 13. DFT calculations for TBZGNR junctions with different width. Configurations and PDOS on edge atoms of 4-ZGNR (a, b) and 8-ZGNR (c, d) with twist angle of 90°. It is clear that in the 4-ZGNR there are some changes on edge states but not very pronounced, while in the 8-ZGNR the edge-states changes are much more abundant than those in 4-ZGNR and in 6-ZGNR, suggesting that the wider ZGNRs will produce more complicated overlapped configurations and more abundant edge states.

Supplementary References:

1 Kawai, S. *et al.* Superlubricity of graphene nanoribbons on gold surfaces. *Science* **351**, 957-961 (2016).

2 van der Lit, J., Jacobse, P. H., Vanmaekelbergh, D. & Swart, I. Bending and buckling of narrow armchair graphene nanoribbons via STM manipulation. *New J Phys* **17,** 053013 (2015).

3 Jung, J. & MacDonald, A. H. Accurate tight-binding models for the pi bands of bilayer graphene. *Phys Rev B* **89**, 035405 (2014).

4 Ruffieux, P. *et al.* On-surface synthesis of graphene nanoribbons with zigzag edge topology. *Nature* **531**, 489-492 (2016).

5 Van Der Lit, J. *et al.* Suppression of electron–vibron coupling in graphene nanoribbons contacted via a single atom. *Nature communications* **4**, 1-6 (2013).

6 Li, Y., Zhang, W., Morgenstern, M. & Mazzarello, R. Electronic and magnetic properties of zigzag graphene nanoribbons on the (111) surface of Cu, Ag, and Au. *Physical Review Letters* **110**, 216804 (2013).

7 Lauwaet, K. *et al.* Resolving all atoms of an alkali halide via nanomodulation of the thin NaCl film surface using the Au (111) reconstruction. *Physical Review B* **85**, 245440 (2012).

8 Blackwell, R. E. *et al.* Spin splitting of dopant edge state in magnetic zigzag graphene nanoribbons. *Nature* **600**, 647-652 (2021).

9 Hu, X., Sun, L. & Krasheninnikov, A. V. Tuning electronic and magnetic properties of zigzag graphene nanoribbons by large-scale bending. *Applied Physics Letters* **100**, 263115 (2012).

10 Levy, N. *et al.* Strain-induced pseudo–magnetic fields greater than 300 tesla in graphene nanobubbles. *Science* **329**, 544-547 (2010).

11 Mao, J. *et al.* Evidence of flat bands and correlated states in buckled graphene superlattices. *Nature* **584**, 215-220 (2020).

12 Wang, G. *et al.* Measuring interlayer shear stress in bilayer graphene. *Physical Review Letters* **119**, 036101 (2017).

13 Li, S.-Y., Su, Y., Ren, Y.-N. & He, L. Valley polarization and inversion in strained graphene via pseudo-Landau levels, valley splitting of real Landau levels, and confined states. *Physical Review Letters* **124**, 106802 (2020).
